# Multimodal Evaluation of Arrhythmogenic Substrate Predicts Atrial Fibrosis and Atrial Fibrillation Recurrence After Catheter Ablation

**DOI:** 10.3390/jcm14186414

**Published:** 2025-09-11

**Authors:** Ioan-Alexandru Minciună, Raluca Tomoaia, Patricia Vajda, Nicoleta Cosmina Hart, Renata Paula Agoston, Tudor Cornea, Georgiana Alexandra Birsan, Andreea-Maria Linul, Gabriel Cismaru, Mihai Puiu, Radu Ovidiu Roșu, Gelu Simu, Dana Pop

**Affiliations:** 1 5th Department of Internal Medicine, Faculty of Medicine, “Iuliu Hațieganu” University of Medicine and Pharmacy, 400347 Cluj-Napoca, Romania; minciuna.ioan.alexandru@elearn.umfcluj.ro (I.-A.M.); hada.nicoleta.cosmina@elearn.umfcluj.ro (N.C.H.); cismaru.gabriel@elearn.umfcluj.ro (G.C.); rosu.radu.ovidiu@elearn.umfcluj.ro (R.O.R.);; 2 Cardiology Department, Rehabilitation Hospital, 400066 Cluj-Napoca, Romania; 3 Department of Electrical Engineering and Measurements, Technical University, 400394 Cluj-Napoca, Romania; patricia.vajda@hellimed.ro

**Keywords:** atrial fibrillation, catheter ablation, P-wave duration, electroanatomical mapping, low-voltage areas, arrhythmogenic substrate, recurrence prediction

## Abstract

**Background/Objectives**: For many years, catheter ablation (CA) has been a cornerstone in atrial fibrillation (AF) rhythm control therapy; however, recurrence remains common. Multiple parameters have been proposed to quantify AF arrhythmogenic substrate, yet reliable predictors of long-term outcomes are lacking. To assess the value of non-invasive amplified P-wave duration (PWD), echocardiographic parameters, biomarkers, and electroanatomical mapping (EAM) were used in predicting left atrial (LA) fibrosis and arrhythmia recurrence after CA. **Methods**: We included 196 patients undergoing first CA for paroxysmal or persistent AF. Amplified 12-lead ECG PWD parameters [Pmax, Pmin and left atrial P-wave (LAP)], echocardiographic parameters, and biomarkers were assessed pre-procedure. We measured low-voltage areas (LVA, 0.2–0.5 mV) on high-density voltage EAM during sinus rhythm as a surrogate of fibrosis. Freedom from arrhythmia was evaluated at 6 and 12 months. **Results**: Patients with LVA on EAM had prolonged Pmax (148 vs. 135 ms, *p* < 0.0001), Pmin (111 vs. 101.5 ms, *p* = 0.0001), LAP (73.5 vs. 55.5 ms, *p* < 0.0001), larger LA diameter (*p* = 0.0002), area (*p* = 0.0365) and volume (*p* = 0.004), higher E/E’ (*p* = 0.0007) and E/A ratios (*p* = 0.037), more mitral regurgitation (*p* = 0.0315), and higher pro-BNP levels (*p* = 0.0094). Univariate analysis showed 12-month recurrence rates higher with greater Pmax, Pmin, LAP, LVA presence and extent; however, in multivariate analysis, only P-wave parameters remained independently associated with recurrence. **Conclusions**: Prolonged PWD parameters strongly reflect LA substrate (Pmax, Pmin) and independently predict post-ablation AF recurrence (Pmax, Pmin, and LAP). LA size, diastolic dysfunction, and mitral regurgitation were associated with LA fibrosis, while pro-BNP was associated with both fibrosis and arrhythmia recurrence. Integrating these simple, non-invasive markers into a multimodal assessment alongside EAM could improve pre-procedural risk stratification and guide individualized ablation strategies.

## 1. Introduction

Atrial fibrillation (AF) is the most common sustained cardiac arrhythmia worldwide and represents a growing global health concern [1]. Catheter ablation (CA), particularly pulmonary vein isolation, has become a cornerstone in the management of AF, promising durable rhythm control. However, the long-term success of CA remains suboptimal, with significant recurrence rates observed during the first year [2,3].

The mechanisms underlying AF recurrence are complex and heterogenous. Three main mechanisms are widely accepted to play a key role in AF pathophysiology: alterations in ion channel activity which lead to changes in atrial depolarization and refractoriness (electrical remodeling), modification of Ca^2+^ handling, leading to atrial contractile dysfunction (contractile remodeling), and finally fibrosis and atrial dilation (structural remodeling) [4,5]. Electrical and contractile remodeling usually occur within minutes from AF onset, whereas structural changes—likely irreversible—appear to develop over periods of weeks to months, explaining the tendency of paroxysmal AF to become persistent (“AF begets AF”) [4,6]. Although these processes are well recognized and define the arrhythmogenic substrate of AF, there is currently no reliable individual predictor of arrhythmia recurrence after CA in clinical practice. Moreover, despite several risk scores integrated parameters to predict AF recurrence after CA, none have shown reproducible long-term predictive performance [7,8,9].

Multiple parameters have been proposed to quantify atrial remodeling and to improve arrhythmia recurrence prediction after CA. Low-voltage areas (LVA) identified using electroanatomical mapping (EAM) are considered surrogates of atrial fibrosis and have been shown by several studies to be associated with less favorable outcomes following CA [9,10,11,12,13]. Surface electrocardiogram (ECG) atrial conduction markers such as P-wave duration (PWD), P-wave dispersion (Pd), and left-atrial P-wave (LAP—measured from −dV/dt in leads V1 and V2 until the end of the P-wave) provide further information on the extent of atrial remodeling [10,11,14,15,16]. Echocardiography offers additional assessment of atrial substrate through measurements such as LA diameter (LAD), area and volume, diastolic function, and degree of mitral regurgitation or left atrial strain, reflecting the degree of atrial remodeling [2,17,18]. Circulating biomarkers including N-terminal natriuretic peptide (pro-BNP) and C-reactive protein (CRP) assessing myocardial stretch and inflammation have also been shown to be related to atrial fibrosis [2,9].

Integrating multiple parameters of atrial remodeling is likely to offer greater predictive value than any single parameter alone. A combined approach that incorporates clinical risk scores, non-invasive ECG and echocardiography parameters, biomarkers, and EAM may have the potential to improve risk stratification and better predict outcomes. In this study we evaluate this comprehensive set of parameters in patients undergoing first radiofrequency (RF) CA for AF. Our goal is to identify the factors most strongly associated with atrial fibrosis and to evaluate their power to predict arrhythmia recurrence over a 12-month follow-up. The broader aim is to support more accurate identification of patients at risk of recurrence, improve selection of candidates for CA, adapt procedural strategies to individual risk profiles, and optimize follow-up care.

## 2. Methods

### 2.1. Patient Population

The study included 196 patients undergoing first RFCA for paroxysmal or persistent AF between 2020 and 2024 at the Clinical Rehabilitation Hospital in Cluj-Napoca. Paroxysmal/persistent AF were defined according to the 2024 European Society of Cardiology Clinical Practice Guidelines for Management of AF [1]. The study was approved by the local ethical committee and all included patients signed written informed consents. We excluded patients without EAM in sinus rhythm (SR) before starting RF applications and re-do procedures. Demographic, clinical (associated comorbidities and drug treatments), biological, electrocardiographic, echocardiographic, and ablation variables were retrospectively assessed. All patients were effectively anticoagulated. Transesophageal echocardiography was performed in all patients prior to CA to exclude LA thrombus.

### 2.2. Acquisition of ECG Parameters

Twelve leads ECGs in standard position were recorded during the CA procedure, prior to RF application start, using the WorkMate Claris System (version 1.2, Abbott, St. Paul, MN, USA). To accurately identify the earliest and latest deflections of the P-wave, a sweep speed of 100–150 mm/s was used and recordings were amplified to 0.2 to 0.25 mV/cm. All PWD measurements were manually performed by two independent cardiologists and included maximum/minimum PWD (Pmax/Pmin) in any of the twelve leads, Pd (Pmax−Pmin) and LAP (measured from −dV/dt in leads V1 and V2 until the end of the P-wave in any of the twelve leads).

### 2.3. Electroanatomical Mapping and Quantification of Low-Voltage Areas

High-density 3D EAM was performed in all patients in SR using a multielectrode mapping catheter (PentaRay NAV, Biosense Webster, Diamond Bar, CA, USA) and CARTO 3 navigation system (version 8.1, Biosense Webster, Diamond Bar, CA, USA). Bipolar electrograms were recorded at 15–250 Hz and displayed at 0.1–0.2 mV/cm. LVA measurement was manually performed by two experienced operators/clinical support engineers using “area measurement” tool and bipolar voltage thresholds of 0.2–0.5 mV. PV ostia and the mitral annulus were excluded from analysis.

### 2.4. Catheter Ablation

CAs were performed by 4 experienced electrophysiologists, under deep sedation, using an irrigated-tip, contact-force sensing catheter (Thermocool SmartTouch SF, Biosense Webster, Diamond Bar, CA, USA). Additionally, a decapolar or quadripolar catheter was used for the coronary sinus. Transseptal puncture was performed under fluoroscopic guidance in all patients. Following access, an intravenous bolus of 5- to 10,000 units of heparin was administered followed by continuous infusion depending on the weight of the patient to achieve activated coagulation times between 300 and 350 ms. RFCA was performed using a high-power, short-duration protocol (50 W, with ablation index between 450 and 500 for the anterior wall and between 350 and 400 for the posterior wall). The primary procedural endpoint was complete electrical isolation of all pulmonary veins, confirmed by entry and exit block. In patients with extensive LA fibrosis, additional posterior box or anterior line was performed, according to LVA localization. Procedural safety was evaluated by intra- and post-procedural complications. Procedural efficiency was assessed by total procedure duration, number of radiofrequency applications, fluoroscopy time, and radiation dose. Total procedure time was defined as from femoral venous puncture to catheter removal from the body.

### 2.5. Echocardiographic Parameters

All patients included in this study performed transthoracic echocardiography using either the Vivid E95 ultrasound system (GE HealthCare, Chicago, IL, USA) or EPIQ ultrasound system (Philips Healthcare, Andover, MA, USA) within 3 days prior to the ablation procedure. With the patient positioned on their left side, routine M-mode and 2D echocardiography was performed. LA anteroposterior diameter was measured in the parasternal long-axis view at end-systole. LA area was measured from the apical four-chamber view, and LA volume was calculated using the biplane area-length method. Diastolic function was assessed according to current guidelines using pulsed-wave Doppler and tissue Doppler imaging. The degree of mitral regurgitation was quantified semi-quantitatively using color Doppler and graded as mild, moderate or severe. Left ventricular ejection fraction (LVEF) was calculated using biplane Simson’s method.

### 2.6. Follow-Up

Patients were discharged after one to three days of observation. The follow-up was performed 6 and 12 months after CA and included clinical cardiology assessment, 12-lead ECG and 24 h Holter monitoring. Antiarrhythmic drugs were maintained for 3 months after CA, representing the blanking period, according to the older 2020 European Society of Cardiology Clinical Practice Guidelines for Management of AF [19]. AF recurrence was defined as any documented (surface or Holter ECG) episode of AF or atrial tachycardia lasting more than 30 s after the blanking period.

### 2.7. Statistical Analysis

Clinical variables were expressed as median [IQR] or as frequencies, depending on the type and distribution of the data. Normality was assessed using the Kolmogorov–Smirnov test. Univariate logistic regression was applied to evaluate the association of fibrosis and AF recurrence with clinical, ECG, echocardiographic, and ablation-related variables within each group. Multivariate logistic regression was then performed to assess whether ECG parameters could independently predict LA fibrosis or AF recurrence while adjusting for clinical covariates using the enter method. Statistical analysis was conducted using MedCalc Statistical Software 19.6.1 (MedCalc Software Ltd., Ostend, Belgium; http://www.medcalc.org, accessed on 10 August 2025). A *p*-value of <0.05 was considered significant.

## 3. Results

### 3.1. Patient Characteristics

The study population consisted of 196 patients who underwent a first treatment of high-power short-duration RFCA for either paroxysmal (127, 64.8%) or persistent AF, with a mean age of 61.28 ± 9.23 years. Most patients were men (123, 62.8%) with preserved left ventricular ejection fraction and 29 patients (14.8%) had ischemic cardiomyopathy. Ninety-one percent were on antiarrhythmic drugs, most of them on Amiodarone. The baseline clinical and biological characteristics of all patients are summarized in Table 1. Nearly one-fifth of the patients (38, 19.4%) demonstrated LA fibrosis, identified as LVA on EAM, most frequently involving the posterior or/and anterior wall, with a median extent of 5.3 cm^2^ (IQR, 3–9.3 cm^2^).

### 3.2. Amplified PWD on 12-Leads Surface ECG Predicts Fibrosis on EAM

**Figure 1:**
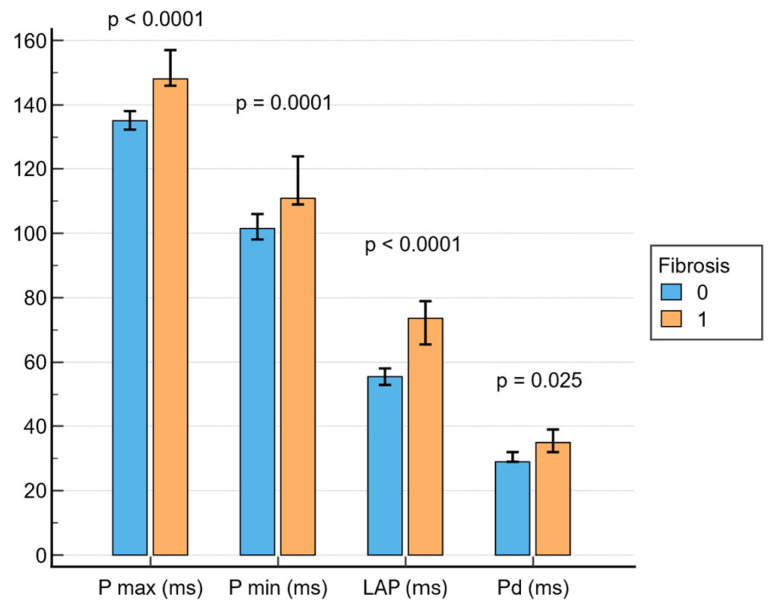
Associations between PWD parameters and fibrosis. LAP = left atrial P-wave.

As shown in Figure 1, patients with LVA on EAM had delayed atrial activation times compared to patients without LA fibrosis on bipolar voltage maps, as revealed by increased Pmax (148 ms; IQR, 143–161 ms vs. 135 ms; IQR, 125–146 ms; *p* < 0.001), Pmin (111 ms; IQR, 105–130 ms vs. 101.5 ms; IQR, 90–117 ms; *p* = 0.001), Pd (35 ms; IQR, 29–40 ms vs. 29 ms; IQR, 24–39 ms; *p* = 0.025) and LAP (73.5 ms; IQR, 59–82 ms vs. 55.5 ms; IQR, 45–66 ms; *p* < 0.001) values. In univariate analysis, all four amplified P-wave markers (Pmax, Pmin, LAP, and Pd) demonstrated significant associations with the presence of LVA on EAM. However, in the multivariate model adjusted for age, AF duration and LA volume, only Pmax and Pmin retained independent associations (Table 2 and Table 3).

### 3.3. Echocardiographic Parameters Predict Fibrosis on EAM

**Figure 2:**
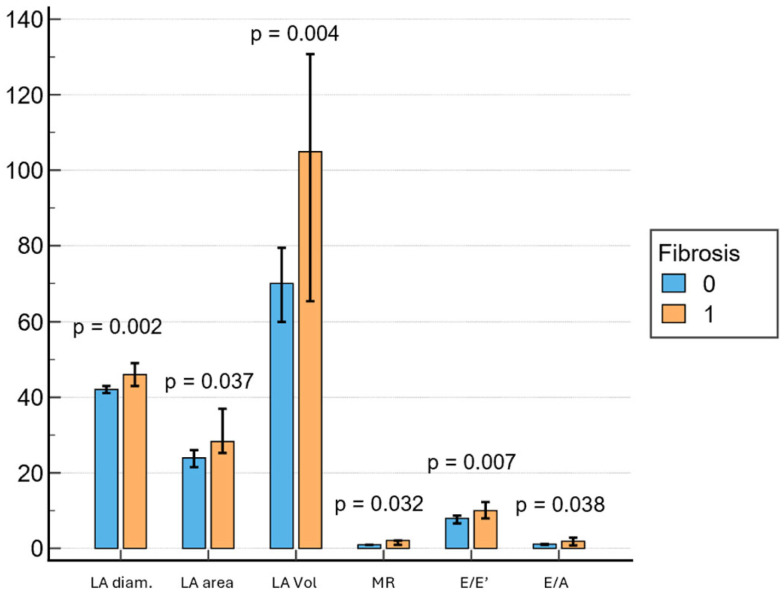
Associations between echocardiography parameters and fibrosis. LA = left atrium, MR = mitral regurgitation.

Subjects with LVA on EAM had significantly larger LA diameter (46 mm; IQR, 41.7–51 mm vs. 42 mm; IQR, 38–45 mm; *p* = 0.002), LA area (28 cm^2^; IQR, 25.2–37 cm^2^ vs. 24 cm^2^; IQR, 20–28.5 cm^2^; *p* = 0.037), LA volume (105 mL; IQR, 62.5–134 mL vs. 70 mL; IQR, 45–89.2 mL; *p* = 0.004), E/E’ (18.0; IQR, 7.6–12.6 vs. 8.0; IQR 6–9.15; *p* = 0.007) and E/A (1.9; IQR, 0.8–2.7 vs. 1.1; IQR 0.8–1.43; *p* = 0.038) (Figure 2). Mitral regurgitation was observed in seventy-four percent of the patients, most frequently of mild severity, and its presence was associated with LVA on EAM (*p* = 0.032).

### 3.4. Procedural and Ablation Parameters Predict Fibrosis on EAM

**Figure 3:**
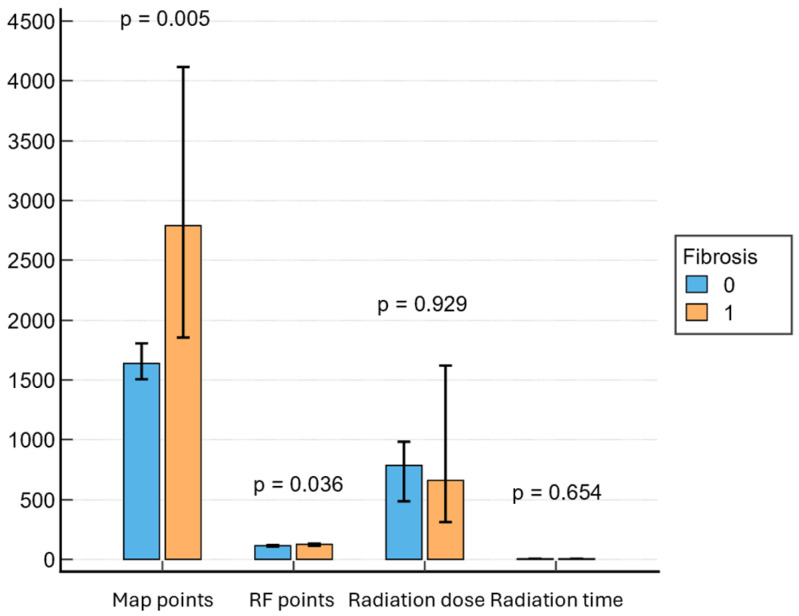
Associations between procedural parameters and fibrosis. RF = radiofrequency.

Patients with LVA on EAM had longer CA procedures (150 min; IQR, 120–180 min vs. 130 min; IQR, 120–160 min; *p* = 0.036) and needed more ablation points (125.47; IQR, 108–157 vs. 114.5; IQR, 94–137; *p* = 0.036). Also, the presence of fibrosis on EAM was associated with a higher probability of requiring electrical cardioversion at the end of the procedure (42.1% vs. 22.2%, *p* = 0.0121). There were no significant differences in terms of radiation exposure between the two groups (Figure 3). Cavotricuspid isthmus ablation for documented atrial flutter was performed in 38 patients (19.4%), followed by box lesion in 26 patients (13.3%). Twenty-three patients (11.7%) needed at least one re-do procedure during the follow-up. A common left pulmonary vein was found in 55 patients (28.1%).

### 3.5. Biomarkers and LVA on EAM

A significant association was found between the presence of LVA on EAM and elevated pro-BNP (*p* = 0.0093) in the univariate analysis, whereas no significant differences were found in terms of CRP, D-dimer or thyroid hormones between the two groups. Moreover, in univariate logistic regression analysis, pro-BNP was also associated with 12-month AF recurrence (*p* = 0.0001).

### 3.6. PWD and LVA on EAM Predict Arrhythmia Recurrence After CA

PWD and the presence and extent of fibrosis on EAM were all predictors for AF recurrence 12 months after CA. Arrhythmia recurrence was significantly higher in patients with higher Pmax (*p* < 0.0001), Pmin (*p* < 0.0001), LAP (*p* < 0.0001), LA LVA on EAM (*p* = 0.0003) and with higher extent of LVA (*p* = 0.0174). In univariate analysis, amplified PWD parameters (Pmax, Pmin and LAP, but not Pd), together with the presence (*p* = 0.0003) and extent of LVA (*p* = 0.0174) on EAM, were significant predictors of AF recurrence after CA. After adjustment for known parameters to be associated with in multivariate analysis, from the previously significant parameters, only Pmax (*p* = 0.0072), Pmin (*p* = 0.0018) and LAP (*p* = 0.0007) retained independent associations (Table 4 and Table 5).

## 4. Discussions

In this study of patients undergoing initial high-power RFCA for AF, we performed a comprehensive, multimodal assessment of the arrhythmogenic substrate. We integrated EAM parameters of LA fibrosis, amplified surface ECG parameters, echocardiography and biomarkers analysis, and evaluated their value in predicting both the presence of LA fibrosis and AF recurrence at 12 months. Several major findings emerge from our data.

First, prolonged PWD on 12-lead surface ECG at baseline predicts the presence of LA fibrosis evaluated by LVA on EAM and is further associated with AF recurrence twelve months after RFCA. Increased values of Pmax, Pmin, and especially LAP (measured from −dV/dt in leads V1 and V2 until the end of the P-wave in any lead) were associated with the presence of fibrosis and predicted post-ablation arrhythmia recurrence. In the univariate analysis for LA fibrosis, all amplified PWD parameters (Pmax, Pmin, Pd and LAP) were associated with LA fibrosis, assessed by LVA on EAM, supporting the concept that atrial conduction delay reflects structural remodeling. However, in the multivariate model adjusted for age, LA volume, and AF duration, only Pmax and Pmin remained independent predictors of LA fibrosis. This is in line with previous works which have confirmed that PWD and interatrial block are predictors of atrial scarring based on EAM data [10,11,14,15,16], cardiac magnetic resonance (CMR) [20,21,22], or histological data [23]. When applied to evaluate AF recurrence 12 months after CA, univariate logistic regression analysis similarly showed that PWD parameters (Pmax, Pmin and LAP), together with the presence and extent of LVA on EAM, are associated with arrhythmia recurrence, with all three parameters retaining independent associations with AF recurrence in the multivariate model. In our study, LAP would have also been expected to retain an independent association with LA fibrosis, not only with arrhythmia recurrence, given that it reflects the interatrial conduction delay and LA conduction time, and has previously been proposed as a marker of atrial scarring [10]. A possible explanation for these results lies in collinearity of LAP with Pmax and Pmin, showing overlapping electrophysiological information, so its predictive contribution may be attenuated with the inclusion of these parameters in the same multivariate model. In addition, LAP may be more sensitive to subtle conduction changes but less robust when adjusted for structural parameters such as LA volume and AF duration. Finally, LAP may be more prone to measurement errors and variability in clinical practice, which makes it more complex and operator-dependent compared to Pmax and Pmin. This suggests that while LAP may be informative and predict LA fibrosis, PWD parameters (Pmax and Pmin) may provide stronger and more independent predictive value of the AF arrhythmogenic substrate in clinical practice.

Despite compelling evidence and the fact that ECG is an inexpensive, simple and widely available tool capable of estimating atrial fibrosis and predicting arrhythmia recurrence, there is still no broadly adopted strategy for incorporating these parameters into clinical decision-making [2,16]. Our findings support the use of amplified P-wave analysis as a simple, non-invasive marker for arrhythmogenic substrate characterization prior to CA. Its impact may improve patient selection, considering that arrhythmia recurrence after CA is markedly higher among patients with LVA [13,16], and can further guide the ablation strategy. Thus, in patients with a higher likelihood of extensive fibrosis, as suggested by prolonged PWD (within a multimodal evaluation), the use of RF may be preferable over cryoablation, since it allows targeting substrate beyond PV isolation.

Secondly, echocardiography, another accessible and widespread tool, can add further discriminatory value. Although LA dimensions, including diameter, area and especially volume, are well-known markers of atrial remodeling and have been associated with AF recurrence after CA [17,24,25], Georgi et al. found no significant relationship between LA indexed volume (LAVI) and AF recurrence. A possible explanation for this may be the very high mean volume values in this cohort [26]. In our study, as expected, all three parameters demonstrated a significant relationship with atrial fibrosis, as identified by LVA on EAM. Moreover, in the univariate analysis, we observed a strong association between diastolic dysfunction, reflected by E/E’ and E/A ratios, and the presence of LVA on EAM. This finding is in line with previous observations by Masuda et al. [27], which found that high E/E’ was an independent predictor of LVA existence and arrhythmia recurrence. Another important echocardiographic marker associated with LVA was mitral regurgitation, which is consistent with previous reports [2]. All these observations support the pathophysiological concept that chronically elevated filling pressures, impaired relaxation and chamber dilation promote fibrotic remodeling, thereby creating a substrate that facilitates AF maintenance and reduces the efficacy of CA. Finally, increasing evidence supports novel parameters, such as LA strain, which may offer superior specificity for substrate characterization in the future [18].

Procedural characteristics also differed between patients with and without fibrosis. Patients with LVA on EAM had longer total procedure times and required a higher number of RF applications, which is consistent with previously published data [11]. Additionally, patients in the fibrosis group were more likely to need electrical cardioversion at the end of the procedure, which reflects the more complex substrate in these patients. There were no differences in terms of radiation exposure between the two groups.

Among the studied biomarkers, only pro-BNP demonstrated a significant association with fibrosis, while other serum markers such as CRP, D-dimer and thyroid function did not show predictive power. This finding supports the role of pro-BNP as a marker of atrial stretch and fibrosis and is consistent with previous reports [2,9]. Recently, Sumiyoshi et al. described in their paper that the atrial cardiomyopathy score, which incorporates E/E’, LAVI, pro-BNP and tricuspid regurgitation peak gradient is associated with LVA on EAM and with AF recurrence [9]. In our study, pro-BNP was the only biomarker significantly associated with AF recurrence at 12 months following CA. Thus, integrating biomarker data into multimodal assessments of the arrhythmogenic substrate represents a feasible and promising approach in the future.

Of note, interpretation of arrhythmia recurrence also requires consideration of postprocedural management. In our study, all patients received antiarrhythmic drug therapy during the blanking period, which may influence early arrhythmia recurrence. Evidence from randomized trials, including EAST-AF and CIRCA-DOSE, showed that the use of antiarrhythmic drugs during the blanking period reduces early recurrences; however, this did not affect 12-month outcomes after CA [28,29,30]. Accordingly, while their use during the blanking period may influence the early recurrence rate, it is unlikely to have affected the 12-month outcomes reported in our study.

### 4.1. Clinical Implications

Taken together, and given the continuous advances in technology in all these fields, our findings support the integration of non-invasive amplified ECG parameters, echocardiography measures, and targeted biomarker analysis with invasive EAM for a more comprehensive evaluation of the AF arrhythmogenic substrate. Several scoring systems have attempted to integrate some of these parameters into clinical practice [2,7,8,9]. To date, none have achieved consistent accuracy in predicting substrate characteristics or ablation outcomes, the existing evidence not being sufficient to support their inclusion in current Society Guidelines [1,2,16].

Among these tools, amplified P-wave parameters can be easily implemented into routine pre-procedural planning using standard 12-lead ECGs. Although the optimal way to do this would be with digital amplification and caliper-based analysis, it can also be achieved more simply by increasing paper speed and voltage gain, allowing a straightforward implementation in most clinical settings. Patients with prolonged PWD could thus be identified as having a higher likelihood of atrial fibrosis and arrhythmia recurrence, which could help individualize clinical approaches based on risk profiles. In addition, substantial progress may come from computational modeling and emerging artificial intelligence, which together with the already available automated P-wave analysis, could offer a more precise and reproducible P-wave assessment and lead to the development of more specific ECG markers for the quantification of the AF arrhythmogenic substrate [2,15].

Ultimately, a multimodal strategy that merges substrate assessment with clinical characteristics should be simple, cost-effective, and most importantly, highly predictive. By identifying electrical markers that independently predict arrhythmia recurrence, our study highlights the potential for a practical, multimodal strategy to improve patient selection, guide procedural approaches, and optimize follow-up, ultimately translating into a more individualized and effective management of AF.

### 4.2. Study Limitations

The main limitation of this study is that it is a single-center, observational, retrospective analysis, which inevitably limits the generalizability of its findings to broader populations and clinical settings. It is therefore not possible to control variations in operator technique and decision-making based on individual patient characteristics. The study population was heterogenous, including both paroxysmal and persistent AF patients, with most of them receiving antiarrhythmic therapy for several weeks prior to the procedure and during the blanking period. However, the use of antiarrhythmic drugs during the blanking period usually affects early but not long-term arrhythmia recurrence. Although measured by two experienced operators/support engineers, fibrosis was not confirmed by CMR or histology. However, bipolar voltage mapping is widely accepted as a surrogate. The follow-up protocol, although standardized, may underestimate asymptomatic recurrences. Further randomized studies with larger sample sizes are necessary to confirm these results and integrate these parameters into a highly predictive multimodal arrhythmogenic substrate evaluation strategy.

## 5. Conclusions

In patients undergoing first CA for AF, prolonged PWD (Pmax and Pmin) on amplified 12-lead surface ECG was strongly associated with atrial fibrosis assessed by LVA on EAM, and independently predicted AF recurrence (Pmax, Pmin and LAP) at 12 months following CA. Among echocardiographic parameters, LA volume, diastolic dysfunction, and mitral regurgitation were associated with fibrosis, while elevated pro-BNP was the only biomarker associated with both fibrosis and arrhythmia recurrence. These findings highlight the value of integrating electrical, structural, and biomarker data for the characterization of AF arrhythmogenic substrate prior to deciding a rhythm control strategy. A multimodal approach that combines amplified surface ECG, echocardiography, and biomarker data alongside intraprocedural bipolar voltage mapping may improve pre-procedural risk stratification and guide procedural decision-making, ultimately improving long-term outcomes.

## Figures and Tables

**Table 1:** Baseline characteristics of patients (N = 196).

Age (years)	61.28 ± 9.23
Male (*n*)	123 (62.8)
Paroxysmal AF (*n*)	127 (64.8)
AF duration (>1 year) (*n*)	179 (91.3)
LVEF < 50% (*n*)	22 (11.2)
LA diameter (mm)	42 (39–47)
LA volume (mL)	73 (51.5–100)
Atrial flutter (*n*)	76 (38.8)
Coronary artery disease (*n*)	29 (14.8)
Hypertension (*n*)	150 (76.5)
Mitral regurgitation (*n*)	145 (74)
Cerebral ischemic event (TIA/stroke) (*n*)	19 (9.7)
Diabetes mellitus (*n*)	30 (15.3)
COPD (*n*)	19 (9.7)
OSA (*n*)	19 (9.7)
LA fibrosis *(n*)	38 (19.4%)

Values are n (%), mean ± SD, or median (interquartile). AF = atrial fibrillation, COPD = chronic obstructive pulmonary disease, LA = left atrium, LVEF = left ventricular ejection fraction, OSA = obstructive sleep apnea, TIA = transient ischemic attack.

**Table 2:** Univariate predictors of atrial fibrosis (N = 196).

Variable	Odds Ratio	95% CI	*p*
Age	1.049	1.004 to 1.095	0.031
AF type	2.472	1.202 to 5.080	0.014
Pmax	1.049	1.027 to 1.072	<0.001
Pmin	1.034	1.014 to 1.054	0.001
Pd	1.034	1.002 to 1.068	0.034
LAP	1.052	1.028 to 1.076	<0.001
AF duration	1.705	0.905 to 3.215	0.099
E/E’ ratio	1.194	1.019 to 1.398	0.030
A	0.070	0.009 to 0.530	0.010
LA volume	1.0221	1.007 to 1.037	0.003
Pro-BNP	3.212	1.333 to 7.738	0.009
D-dimer	0.994	0.982 to 1.006	0.3513
First-pass PV isolation	1.158	0.562 to 2.387	0.690

AF = atrial fibrillation, LA = left atrium, LAP = left atrial P-wave, Pd = P-wave dispersion (Pmax−Pmin), PV = pulmonary veins.

**Table 3:** Multivariate predictors of atrial fibrosis (N = 196).

Variable	Odds Ratio	95% CI	*p*
Pmax	1.041	1.007 to 1.077	0.017
Pmin	1.039	1.007 to 1.072	0.016
Pd	1.030	0.997 to 1.064	0.071
LAP	0.997	0.942 to 1.055	0.916

LAP = left atrial P-wave, Pd = P-wave dispersion (Pmax−Pmin).

**Table 4:** Univariate predictors of AF recurrence (N = 196).

Variable	Odds Ratio	95% CI	*p*
AF type	1.103	0.611 to 1.991	0.745
Pmax	1.039	1.021 to 1.058	<0.001
P min	1.036	1.019 to 1.054	<0.001
Pd	1.006	0.980 to 1.032	0.664
LAP	1.046	1.025 to 1.067	<0.001
LVA on EAM	4.232	1.955 to 9.159	0.001
LVA extent	1.125	1.021 to 1.239	0.017
LVA localization	3.879	1.144 to 13.159	0.030
First-pass PV isolation	0.775	0.437 to 1.372	0.381
Pro-BNP	3.722	1.961 to 7.064	0.001

AF = atrial fibrillation, EAM = electroanatomical mapping, LAP = left atrial P-wave, LVA = low-voltage areas, Pd = P-wave dispersion (Pmax−Pmin), PV = pulmonary veins.

**Table 5:** Multivariate predictors of AF recurrence (N = 196).

Variable	Odds Ratio	95% CI	*p*
Pmax	1.040	1.010 to 1.070	0.007
P min	1.046	1.017 to 1.077	0.002
Pd	0.973	0.932 to 1.017	0.223
LAP	1.059	1.025 to 1.095	0.001

LAP = left atrial P-wave, Pd = P-wave dispersion (Pmax−Pmin).

## Data Availability

Data supporting the findings of this study are available from the corresponding author upon reasonable request.
